# The Institutional Library

**Published:** 1915-12-04

**Authors:** 


### THE INSTITUTIONAL LIBRARY.

### THREE WAR PRIMERS.

**Cerebro-Spinal Fever.** By T. J. Horder, M.D. Price 3s. 6d.

**Injuries of the Eyes, Nose, Throat, and Ears.** By A. Maitland Ramsay, M.D.; J. Dundas Grant, M.D.; H. Lawson Whale, M.D.; and C. E. West, F.R.C.S. Price 2s. 6d.

**Medical Hints for Medical Officers Employed with Troops.** By J. E. Squire, M.D., C.B. Price 2s. 6d.

(London: Henry Frowde and Hodder and Stoughton. 1915.)

The first of these three " War Text-books " is a monograph which might just as well have appeared in civilian guise, for there is nothing essentially military in its subject-matter. Major Horder was probably wearing khaki when he gathered some of the experience which he now collates for general information, but he must have been a civilian when most of his knowledge of the subject was acquired. The book summarises in handy form pretty well everything that is commonly accepted about this curious disease: wisely, we think, there is no attempt to be encyclopædic or to enter into controversial points. The sections that are most likely to be criticised are those dealing with the serum treatment, which has disappointed so many British physicians during the recent epidemic, but is warmly recommended by Dr. Horder: either the latter's experience has been more fortunate than that of his colleagues or he is relying too much upon American statistics which have not been borne out by British experience. There is a description of the commoner forms of aberrant cerebro-spinal fever, but it does not include any clinical varieties quite like those published recently in this journal (see The Hospital, September 18, p. 521), which Dr. Horder might perhaps not be disposed to regard as indisputably cerebro-spinal fever at all.

In the second of these war primers is contained a clear exposition of the treatment of injuries of the organs of special sense which should prove of great service to surgeons both at the Front and at the Base in guiding their procedures. Dr. Ramsay is in favour of prophylactic anti-tetanus serum injection when there is reason to suspect the presence of tetanus bacilli; he might add, but does not, that this includes every wound, however caused and wherever situated, sustained in modern warfare. We are glad to see that he scouts the idea, industriously propagated in the lay Press, and even countenanced by some medical practitioners who ought to know better, that amblyopia can arise by "shell concussion" otherwise than as a functional (hysterical) manifestation; there has been far too much nonsense talked lately about physical injuries caused by the wind of a passing shell, and so forth. We are glad to see also that Drs. Grant and Whale discriminate between such methods of treatment as are advisable in medical units at the Front and those which should rather be attempted at the Base. We see no information upon nyctalopia, which last winter was responsible for a large number of transfers from the Front to the Base; most of the patients, but not all, were malingering, and a little advice as to the separation of the true from the false cases would be of value to junior officers. Altogether a most valuable book for medical officers abroad.

Dr. Squire's book is less useful than the other two, as it is almost wholly a brief synopsis of those medical diseases which a medical officer is most likely to be called upon to treat. Indeed, it is so brief as to be useless to any practitioner worthy of being on the register, and is at best a somewhat blind guide; the section on enteric fevers, the most important of all in a book of this aim and scope, is scrappy and feeble to a degree, and omits many things about which a junior medical officer might expect to find expert help and advice. Judging purely from internal evidence, one would guess that this author has not had the advantage of serving abroad (in this campaign), and that if he had he would have compiled a better primer.

**A Surgeon in Khaki.** By Arthur Anderson Martin, M.D., Ch.B., F.R.C.S. Ed. With illustrations. (London: Edward Arnold. 1915. Price 10s. 6d. net.)

Among the books about the war which doctors have hastened to supply on the encouragement of eager publishers, Dr. Martin's has the interest of being one written from the point of view of a Colonial, who had already seen active service during the South African War. It is a simple, straightforward account of what he has been through, which reads as though his diary had coalesced into the various chapters of this volume. He happened to be at Aberdeen during a professional tour when war was declared, whereupon he at once offered his services to the Royal Army Medical Corps, in which he received a temporary commission. After some dreary days at Aldershot, he crossed to France to begin work at one of the bases of the Expeditionary Force. His description of the confusion that everywhere prevailed at that time gives a touch of actuality often lacking in the more romantic adventures of those who have been taken prisoners or otherwise were inveigled into the debateable ground between the two armies, which varied from day to day in the course of the German advance. After this experience at the Base, Dr. Martin moved to the Front, which he reached at the moment of the Allied offensive. His account of the movements of a field ambulance across France to Flanders gives an unembroidered description of marches by day and by night, camping at chateaux, of operating under improvised conditions, and of such excitements on the road as were afforded by rumours, orders to proceed to secret destinations, and so forth.

The criticisms and reflections are not numerous, and refer mainly to minor points, such as the officers' khaki cap which, he says, affords no protection from the heat, and soaks up the rain like a sponge. Like everyone else who experienced the lack of them in the early fighting, Dr. Martin reiterates "the infinite and lasting benefit" which the motor ambulances have afforded. He cannot understand why there were none at the outset of the war, and declares that five years ago he could have told any inquirer that the best way of transporting the sick and wounded in the field was by motor ambulance. The brief glance he allows us of the ravages of "gas gangrene," the effects of shell-shock, and of the piteous condition to which the sound of firing reduces many of the wounded, gives a cruel picture of the machine-made brutality of modern war. In this connection we may quote what Dr. Martin describes as "the only certain proof of the employment of Dum-dum bullets"—namely, the finding of them in the enemy's trenches—as reminding us that the destruction accompanying certain bullet wounds is not sufficient to show that "Dum-dum" bullets have been used. He adds, however, that the Germans in Togoland and in France have been proved to have used bullets with a soft core—in short, of an expansive type prohibited by the Hague Convention. Among his general reflections is one which has been made repeatedly by similar writers—namely, the value of the chaplains and the religious sisters, for all of whom he expresses both admiration and respect. The latter, he says, are beloved by the French soldier, and this remark leads him to contrast the "serious religious note" in the letters of French soldiers with its absence in those written by the British. As it was the author's office on one occasion to be censor of the letters written by the wounded in a clearing hospital, he should be in a position to know the facts on this head. The best chapter in the book is that entitled "Over the Belgian Frontier," which gives a lively and convincing description of the situation in and around Ypres in the course of the city's bombardment. Intended for the general public, and embodying a matter-of-fact account of things seen, "A Surgeon in Khaki" is sure of a welcome by many readers.

**Tropical Hygiene.** By Surgeon-General Sir Pardey Lukis, K.C.S.I., and Lieut.-Colonel R. J. Blackham, C.I.E. Third Edition. (Calcutta: Thacker, Spink, and Co. Pp. 302. Price 3 rupees.)

The preface to this reissue of a manual which is very popular in the East emphasises that the aim which the authors have in view is the instruction of the European resident in the Tropics. They do address also the junior practitioner and the student of medicine, but in the main their appeal is to a lay audience. As long as this is clearly understood, there can be no doubt that the book serves a most valuable purpose, and deserves the rapid success it has attained. It necessarily omits much that the doctor practising in the Tropics ought to know, yet few even of expert tropical hygienists could read it without learning something fresh and worth knowing. It is not too much to say that every company commander in India—and for that matter every company sergeant-major—should possess a copy of this book, and should put in practice its teachings in dealing with the interior economy of his company.

